# Supplementary material for: A Risk–Benefit Analysis of First Nation’s Traditional Smoked Fish Processing
Source: Foods. 2022 Dec 26;12(1):111. doi: 10.3390/foods12010111 (PMC9818569; doi:10.3390/foods12010111)
Supplement: Supplementary file 1 [file foods-12-00111-s001.zip › foods-2060351-supplementary.pdf]

foods

Supplementary Material

A risk-benefit analysis of First Nation's traditional-smoked fish processing

David D. Kitts [1]*, Anubhav Pratap Singh [1], Anika Singh [1], Hao Jing [1], Xiumin Chen [1,2] and Siyun Wang [1]

1 Food Science, Food Nutrition and Health, Faculty of Land and Food Systems, the University of British Columbia, Vancouver, B.C. V6T 1Z4, Canada
2 School of Food and Biological Engineering, Jiangsu University, Jiangsu, Zhenjiang 212013, China
* Correspondence: david.kitts@ubc.ca; Tel.: +1 (604)-822-5560.

Table S1. Toxicity parameters for polycyclic aromatic hydrocarbons (PAHs) used to calculate the Toxic equivalent factors for total Benzo[a]pyrene (BaP).

PAH	M.W.	TEF [1]	EPA [2] Classification
Naphthalene	128.2	-	-
Acenaphthylene	152.2	0.001	D
Acenaphthene	154.2	0.001	-
Fluorene	166.2	0.001	D
Phenanthrene	178.2	0.001	D
Anthracene	178.2	0.01	D
Fluoranthene	202.3	0.001	D
Pyrene	202.1	0.001	D
Benzo(a)anthracene	228.3	0.1	B2
Chrysene	228.3	0.01	B2
Benzo(b)fluoranthene*	252.3	0.1	B2
Benzo(k)fluoranthene*	252.3	0.1	B2
Benzo(a)pyrene*	252.3	1	B2
Indeno(1,2,3-cd)pyrene*	276.3	0.1	B2
Dibenz(a,h)anthracene*	278.3	5	B2
Benzo(g,h,i)perylene*	276.4	0.01	D

1 TEF: toxicity equivalency factor.
2 EPA: United States Environmental Protection Agency; D: not classifiable as to human carcinogenicity; B2: probable carcinogen; evidence from animal studies but inadequate evidence from human studies.
* not identified in smoked fish in our study.

Table S2. Complete fatty acid composition of First Nation's non-smoked and smoked salmon [1].

Fatty acid	First Nation Salmon			Commercial Salmon
	Non smoked	Half smoked	Fully smoked	Smoked
C12 Lauric	< 0.001	0.006 ± 0.001	0.007 ± 0.006	0.005 ± 0.003
C14 Myristic	0.581 ± 0.062	0.389 ± 0.058	0.437 ± 0.383	0.233 ± 0.037
C14:1 Myristoleic	0.016 ± 0.003	0.009 ± 0.001	0.007 ± 0.006	< 0.001
C16 Palmitic	2.471 ± 0.219	1.616 ± 0.199	1.767 ± 0.931	1.337 ± 0.204
C16:1 Palmitoleic	1.152 ± 0.286	0.446 ± 0.057	0.434 ± 0.318	0.288 ± 0.055
C18 Stearic	0.548 ± 0.070	0.339 ± 0.042	0.378 ± 0.163	0.294 ± 0.062
C18:1trans	0.038 ± 0.040	0.023 ± 0.02	0.023 ± 0.012	0.026 ± 0.008
C18:1 Oleic	3.759 ± 0.632	2.403 ± 0.307	2.688 ± 1.722	1.284 ± 0.117
C18:2 trans	0.056 ± 0.005	0.032 ± 0.004	0.037 ± 0.021	0.038 ± 0.011
C18:2 Linoleic	0.445 ± 0.093	0.212 ± 0.027	0.240 ± 0.181	0.112 ± 0.001
C18:3 Linolenic	0.338 ± 0.081	0.126 ± 0.015	0.162 ± 0.142	0.123 ± 0.048
C18:3 Gamma Linolenic	0.024 ± 0.006	0.008 ± 0.001	0.008 ± 0.006	0.007 ± 0.000
C18:4 Moroctic	0.303 ± 0.035	0.137 ± 0.021	0.180 ± 0.199	0.104 ± 0.015
Conjugated Linoleic	0.033 ± 0.003	0.028 ± 0.004	0.031 ± 0.018	0.023 ± 0.006
C20 Arachidic	0.025 ± 0.002	0.019 ± 0.002	0.018 ± 0.013	0.011 ± 0.003
C20:1 Eicosenoic	1.874 ± 0.237	2.045 ± 0.265	1.981 ± 1.218	0.519 ± 0.290
C20:2 Eicosadienoic	0.080 ± 0.009	0.050 ± 0.006	0.057 ± 0.051	0.047 ± 0.008
C20:3 Eicosatrienoic	0.103 ± 0.019	0.048 ± 0.005	0.057 ± 0.046	0.036 ± 0.008
C20:4 Arachidonic	0.200 ± 0.058	0.054 ± 0.004	0.061 ± 0.035	0.060 ± 0.017
C20:5 W3 (EPA)	1.178 ± 0.087	0.721 ± 0.068	0.775 ± 0.592	0.731 ± 0.093
C22:0 Behenic	0.013 ± 0.001	0.007 ± 0.001	0.005 ± 0.003	0.007 ± 0.001
C22:1 Erucic	1.607 ± 0.222	1.699 ± 0.224	1.779 ± 1.167	0.498 ± 0.025
C22:2 Docasadienoic	0.017 ± 0.001	0.010 ± 0.001	0.012 ± 0.008	0.009 ± 0.001
C22:4 Docosatetraenoic	0.058 ± 0.019	0.010 ± 0.001	0.011 ± 0.008	0.010 ± 0.002
C22:5 Docosapentaenoic	0.489 ± 0.063	0.257 ± 0.028	0.313 ± 0.239	0.185 ± 0.025
C22:6 (DHA)	2.213 ± 0.172	1.316 ± 0.120	1.224 ± 0.699	1.698 ± 0.250
C24:0	0.008 ± 0.001	0.004 ± 0.000	0.003 ± 0.001	0.005 ± 0.001
C24:1 Nervonic	0.137 ± 0.016	0.136 ± 0.015	0.137 ± 0.062	0.060 ± 0.016

[1] Values represent mean ± SD expressed as (g/100g). No statistical comparison was made.
